# Bronchial Atresia Presenting With Recurrent Pneumonia in an Adult With Asthma: The Importance of Post-infectious Radiological Reassessment

**DOI:** 10.7759/cureus.102738

**Published:** 2026-01-31

**Authors:** Gonçalo Carneiro, Rita Aranha, Bernardo Macedo

**Affiliations:** 1 Internal Medicine, Unidade Local de Saúde (ULS) de Entre Douro e Vouga, Santa Maria da Feira, PRT; 2 Oncology, Unidade Local de Saúde (ULS) de Entre Douro e Vouga, Santa Maria da Feira, PRT

**Keywords:** bronchial atresia, congenital bronchial anomaly, covid-19, recurrent pneumonia, regional emphysema

## Abstract

Congenital bronchial atresia (CBA) is a rare developmental anomaly characterized by focal obliteration of a segmental bronchus. It is typically asymptomatic and discovered incidentally; however, symptomatic presentations with recurrent respiratory infections have been reported, particularly in pediatric populations.

We report a 39-year-old woman with well-controlled asthma who developed recurrent left lower lobe pneumonia following COVID-19 infection. Despite appropriate antibiotic therapy, she required hospitalization due to clinical recrudescence. Initial computed tomography (CT) demonstrated active inflammatory changes, while reassessment CT after clinical resolution revealed a mucus-filled, finger-like structure extending from the hilum without communication with the bronchial tree, along with regional emphysema, findings consistent with bronchial atresia of the left lower lobe, an atypical location. Conservative management was adopted following multidisciplinary discussion. This case highlights the importance of considering structural bronchial anomalies in recurrent localized pneumonia and demonstrates that bronchial atresia may present symptomatically in adults with unusual anatomical distributions, reinforcing the value of appropriately timed post-infectious radiological reassessment.

## Introduction

Congenital bronchial atresia (CBA) is a rare developmental anomaly characterized by focal obliteration of a segmental or subsegmental bronchus with normal distal bronchial development. During normal embryogenesis, the respiratory system originates from the respiratory diverticulum (lung bud), which appears around the fourth week of gestation as a ventral outgrowth of the foregut endoderm and subsequently undergoes progressive branching to form the tracheobronchial tree [1]. First described in 1953, CBA results from interruption of bronchial continuity during fetal development, leading to mucus accumulation within the blind-ending bronchus (bronchocele) and distal parenchymal hyperinflation via collateral ventilation [2,3]. The etiology remains incompletely understood, though proposed mechanisms include loss of connection between proliferating cells and the developing respiratory bud, possibly secondary to focal ischemia after the 16th week of gestation or traumatic events during fetal life [4,5].

Bronchial atresia demonstrates an anatomical predilection, with involvement of the apicoposterior segment of the left upper lobe in approximately 50% of cases, followed by the right upper lobe (20%) and the lower lobes (15% each) [6,7]. However, these distributions are derived from published case series and may be influenced by ascertainment bias, as patients with symptomatic or atypical presentations are more likely to undergo imaging and be reported. The condition shows a slight male predominance, with an estimated prevalence of 1.2 cases per 100,000 [8]. Clinically, 50%-70% of patients remain asymptomatic, with incidental detection most commonly occurring in the second or third decade of life [9,10]. Symptomatic presentations include dyspnea (14%), recurrent respiratory infections (up to 21%), cough (6%), and less frequently chest pain, hemoptysis, or spontaneous pneumothorax [11,12]. Given the predominantly incidental nature of diagnosis, the true prevalence and anatomical distribution of CBA in the general population remain uncertain.

Computed tomography (CT) is the diagnostic modality of choice, demonstrating characteristic features such as a branching tubular or nodular opacity radiating from the hilum (the mucus-filled bronchocele with a “finger-in-glove” appearance), regional pulmonary hyperlucency due to air trapping and emphysematous change, and hypovascularity of the affected segment [13]. Management ranges from conservative surveillance in asymptomatic patients to surgical resection (segmentectomy or lobectomy) in cases of recurrent severe infection, refractory symptoms, or when malignancy cannot be confidently excluded [14].

We present a case of bronchial atresia with an atypical left lower lobe location, manifesting as recurrent pneumonia in an adult with underlying asthma following COVID-19 infection. This case highlights the importance of systematic radiological reassessment after pneumonic episodes.

This article was previously presented as a poster at the 2022 Internal Medicine National Congress, organized by the Portuguese Internal Medicine Society (October 2022).

## Case presentation

A 39-year-old Caucasian woman with a medical history of bronchial asthma and allergic rhinitis presented to our emergency department (ED) with recurrent respiratory symptoms. She had been followed regularly in the immunoallergy outpatient clinic for well-controlled asthma, maintained on combination therapy with budesonide/formoterol (160 μg/4.5 μg, two inhalations twice daily) and montelukast (10 mg once daily at bedtime). Prior to this presentation, she had never required hospitalization for asthma exacerbations. She was a non-smoker with no history of occupational or environmental exposure to respiratory irritants. Her family history was non-contributory for respiratory diseases.

**Figure 1:**
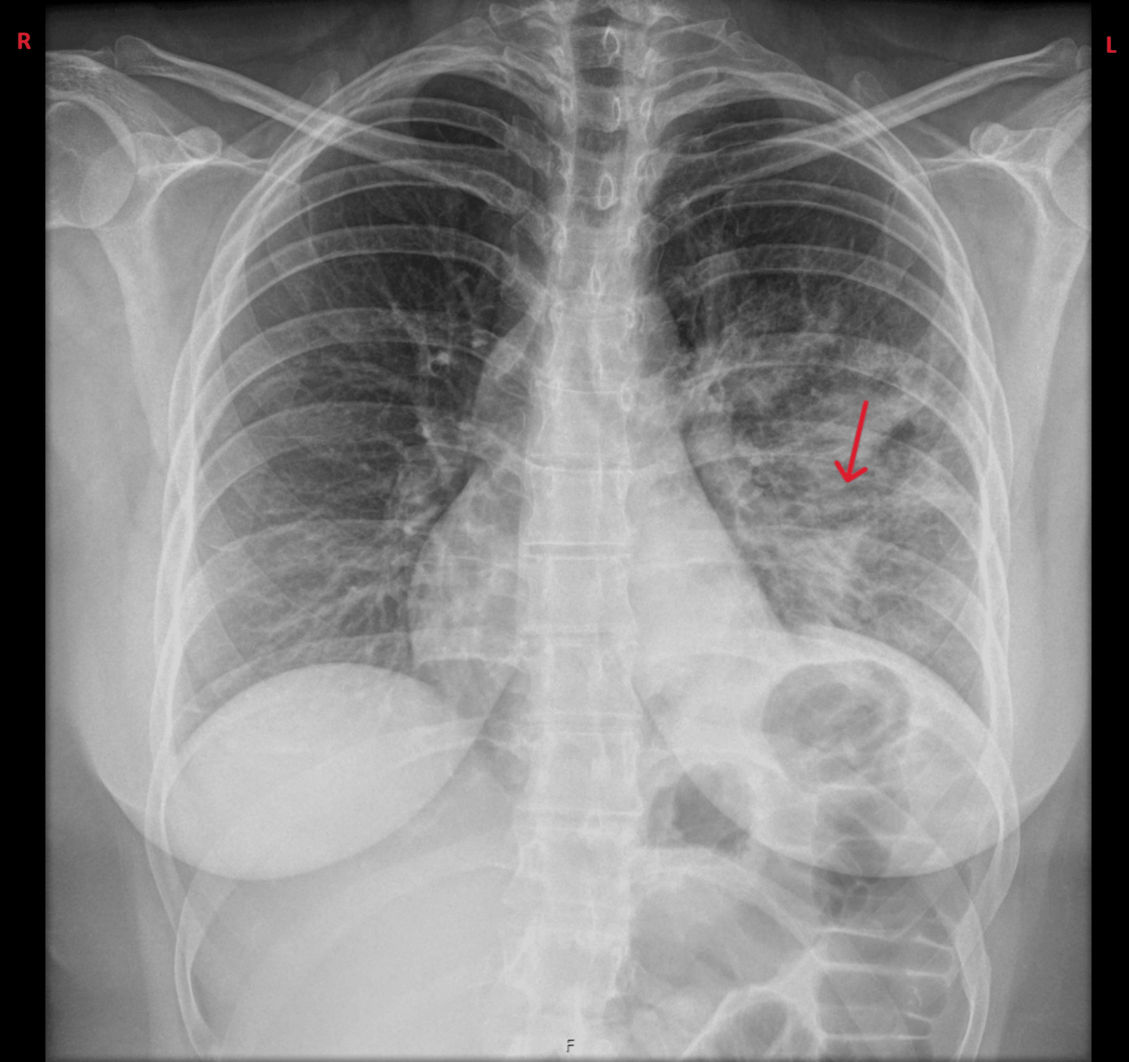
Chest radiograph showing left lower lobe consolidation consistent with pneumonia (red arrow).

In January 2022, the patient was diagnosed with COVID-19, experiencing mild symptoms that resolved with symptomatic treatment and without hospitalization. Two weeks after COVID-19 diagnosis, she developed recurrence of respiratory symptoms and fever, prompting consultation with her primary care physician, who prescribed systemic corticosteroid therapy with prednisolone 20 mg daily and a five-day course of azithromycin. The patient improved clinically, but one week later experienced symptom recrudescence. She presented to the ED of another hospital, where chest radiography demonstrated left lower lobe consolidation (Figure 1).

She was treated with amoxicillin-clavulanate (875 mg/125 mg) for eight days and azithromycin for three days, with initial improvement. However, two weeks following this episode, she developed fever, dyspnea, productive cough, and presented to our ED. On presentation, vital signs were heart rate 120 beats per minute, respiratory rate 21 breaths per minute, blood pressure 155/83 mmHg, tympanic temperature 38.1°C, and oxygen saturation 99% on room air. Heart rate normalized following antipyretic administration and defervescence. Physical examination revealed coarse crackles in the left lower lung field on auscultation; the remainder of the examination was unremarkable, with no signs of respiratory distress, cyanosis, or clubbing. Arterial blood gas analysis on room air showed pH 7.49, pCO₂ 29 mmHg, pO₂ 91 mmHg, Na⁺ 136 mmol/L, K⁺ 3.9 mmol/L, glucose 137 mg/dL, lactate 2.1 mmol/L, and HCO₃⁻ 22.1 mmol/L. Laboratory and microbiological investigations are summarized in Table 1.

**Table 1:** Laboratory and microbiological investigations at hospital admission AST: aspartate aminotransferase, ALT: alanine aminotransferase, GGT: gamma-glutamyl transferase, HIV: human immunodeficiency virus.

Parameter	Result	Reference range
Hemoglobin	12.5 g/dL	12.0-16.0 g/dL
White blood cells	14.3 × 10⁹/L	4.0-11.0 × 10⁹/L
Neutrophils	8.4 × 10⁹/L	2.0-7.5 × 10⁹/L
Platelets	187 × 10⁹/L	150-400 × 10⁹/L
C-reactive protein	98 mg/L	<5 mg/L
Procalcitonin	0.58 ng/mL	<0.5 ng/mL
Creatinine	0.6 mg/dL	0.5-1.2 mg/dL
Urea	38 mg/dL	15-45 mg/dL
Sodium	144 mmol/L	136-145 mmol/L
Potassium	4.1 mmol/L	3.5-5.0 mmol/L
AST	26 U/L	5-40 U/L
ALT	28 U/L	5-40 U/L
GGT	32 U/L	5-55 U/L
Alkaline phosphatase	50 U/L	30-120 U/L
Immunoglobulin A (IgA)	214 mg/dL	65-421 mg/dL
Immunoglobulin G (IgG)	670 mg/dL	552-1631 mg/dL
Immunoglobulin M (IgM)	86 mg/dL	33-293 mg/dL
HIV-1/2 antibodies and p24 antigen	Negative	-
Arterial blood gas		
pH	7.49	7.35-7.45
pCO₂	29 mmHg	35-45 mmHg
pO₂	91 mmHg	60-108 mmHg
HCO₃⁻	22.1 mmol/L	21-28 mmol/L
Lactate	2.1 mmol/L	<2 mmol/l
Microbiological studies		
Sputum culture	No significant pathogen	-
Blood cultures (×2)	Negative at five days	-
Urinary antigen (*Streptococcus pneumoniae*)	Negative	-
Urinary antigen (*Legionella pneumophila*)	Negative	-
Respiratory virus molecular panel	Negative	-

**Figure 2:**
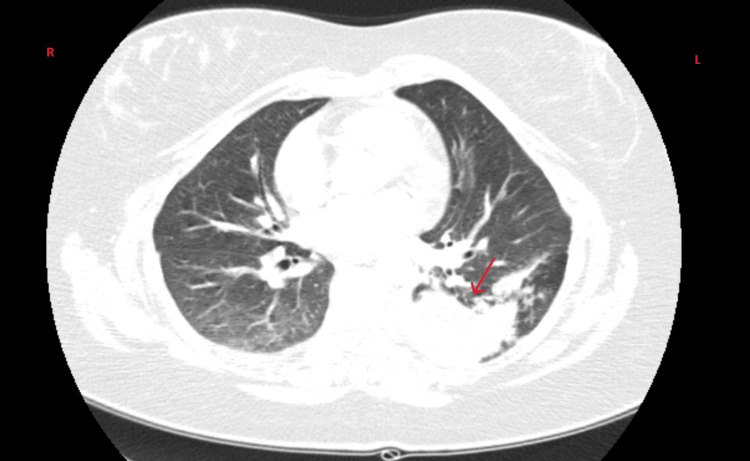
Computed tomography of the chest at emergency department presentation, showing ground-glass opacification and consolidation of the left lower lobe (red arrow), consistent with an acute inflammatory/infectious process (pneumonia). The underlying bronchial atresia is not readily apparent due to active inflammation.

Chest CT revealed a patent pulmonary arterial tree without pulmonary embolism. Irregular densification was observed in the superior segment of the left lower lobe and in the posterobasal and laterobasal segments, with ill-defined borders suggesting an inflammatory/infectious pneumonic process, though non-specific. Post-therapeutic imaging reassessment was recommended to exclude a potential neoplastic lesion. No mediastinal lymphadenopathy, well-defined pulmonary nodules, or ground-glass opacities were identified (Figure 2).

Given the clinical and radiologic evidence of treatment failure (persistent symptoms with fever, dyspnea, and cough despite completed outpatient antibiotic course, laboratory findings showing neutrophilic leukocytosis and elevated C-reactive protein, and CT demonstrating ongoing inflammatory process in the left lower lobe), the patient was admitted to the internal medicine ward and initiated on broad-spectrum intravenous antibiotic therapy with piperacillin-tazobactam (4.5 g every eight hours). During hospitalization, fiberoptic bronchoscopy was performed to exclude endobronchial obstruction, foreign body, or malignancy. The procedure revealed no structural pulmonary abnormalities or suspicious neoplastic lesions; the bronchial tree appeared anatomically normal up to the visible subsegmental bronchi. Tracheal aspirate was collected for sputum culture and mycobacterial culture, and bronchoalveolar lavage was performed for mycobacterial DNA detection by molecular biology; all these studies were negative. Also, evaluation for predisposing factors revealed no evidence of major risk factors for aspiration (no dysphagia, neurological impairment, or sedating medications) or immunosuppression (negative HIV serology, serum immunoglobulin levels within normal range (as shown in Table 1), no malignancy identified, and no immunosuppressive therapy or chronic systemic corticosteroid use beyond standard inhaled treatment for asthma). The patient demonstrated favorable clinical evolution with defervescence within 48 hours, progressive improvement in dyspnea, and resolution of cough. After three days on the general ward with continued clinical stability, she was transitioned to home hospitalization services to complete an eight-day course of intravenous antibiotic therapy. She was discharged with complete symptom resolution and scheduled for follow-up.

**Figure 3:**
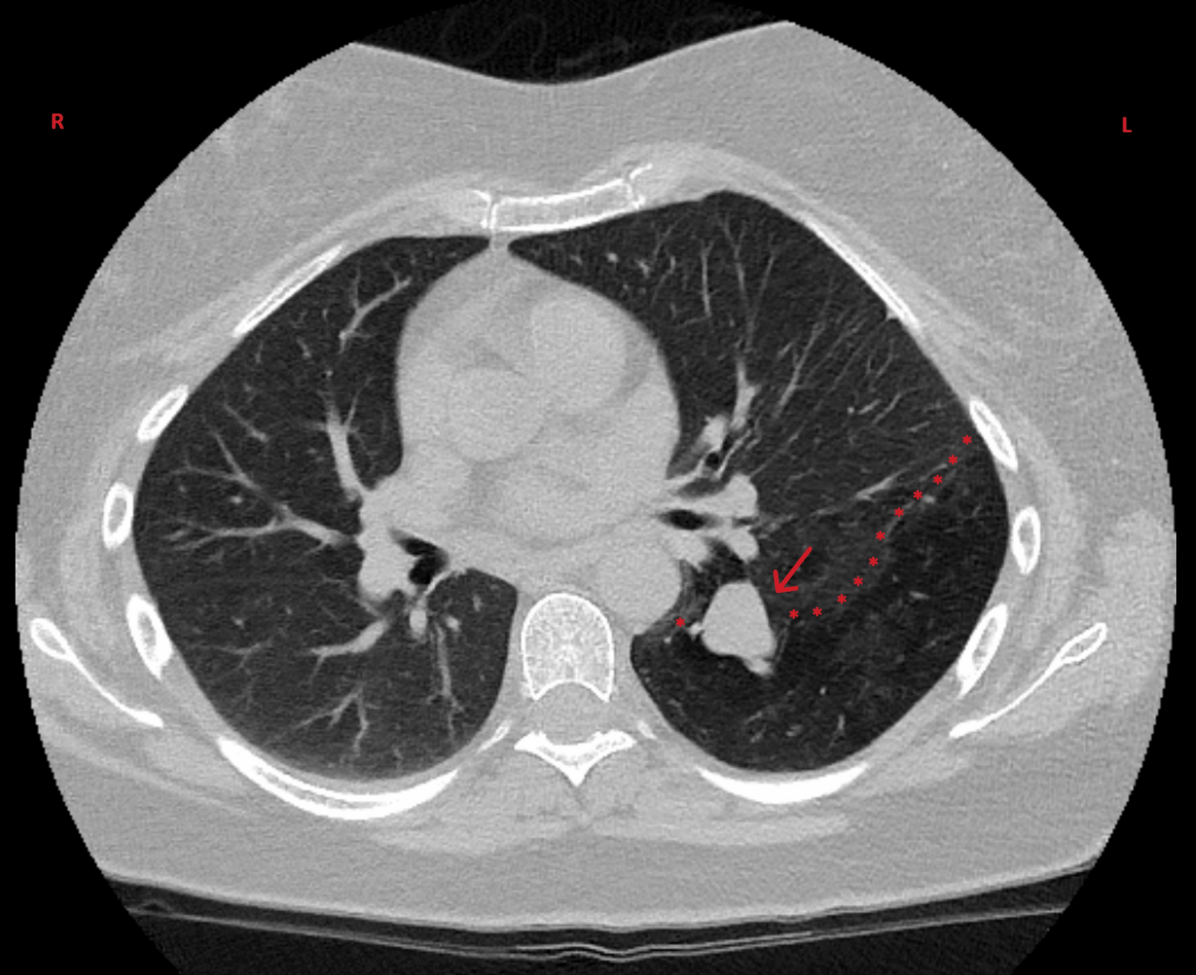
Follow-up computed tomography of the chest three weeks after clinical resolution, revealing a finger-like mucus-filled structure extending from the left hilum (red arrow), measuring up to 13 mm in maximum diameter, without communication to the remaining bronchial tree. Adjacent parenchyma demonstrates marked emphysematous changes (red stars). These findings are characteristic of congenital bronchial atresia. Note the complete resolution of previous consolidation, confirming the inflammatory/infectious nature of the initial findings.

Three weeks following discharge, the patient was evaluated in the internal medicine outpatient clinic, remaining completely asymptomatic. Repeat chest CT demonstrated complete resolution of the previously identified consolidation, confirming its inflammatory/infectious nature. However, the study revealed a finger-like structure extending from the left hilum with a maximum caliber of 13 mm, corresponding to mucus-filled bronchi without communication with the remaining tracheobronchial tree. The adjacent lung parenchyma in the left lower lobe demonstrated marked emphysematous changes with anterior displacement of the left upper lobe secondary to hyperinflation of the affected segment. These findings were highly suggestive of congenital bronchial atresia (Figure 3).

The case was discussed in a multidisciplinary meeting with the pulmonology department. Given the patient's excellent clinical response, complete symptom resolution, and the benign nature of the anatomical finding, a conservative management approach was adopted. The patient was counseled regarding the diagnosis and warning signs warranting urgent evaluation. She was transitioned from immunoallergy to pulmonology follow-up for ongoing asthma management. During subsequent follow-up extending over 18 months, she experienced minor respiratory infections that resolved with conservative management, without requiring hospitalization. Her asthma remained well-controlled on her baseline regimen.

Written informed consent was obtained from the patient for publication of this case report, including clinical data and imaging studies.

## Discussion

As described earlier, bronchial atresia results from developmental disruption of the normally progressive branching morphogenesis of the tracheobronchial tree [1]. In contrast to normal lung development, where bronchial continuity is maintained throughout gestation, bronchial atresia involves focal interruption of an already-formed segmental or subsegmental bronchus while distal structures continue to develop normally. Several theories attempt to explain this paradoxical pattern. The most widely accepted hypothesis proposes focal vascular insufficiency after the 16th week of gestation, a critical period when the bronchial tree has already undergone significant branching, leading to ischemic obliteration of a specific bronchial segment [2,3]. Alternative theories include in utero traumatic events or loss of proliferative connection between developing airway buds, though these remain less substantiated. What distinguishes bronchial atresia from other congenital pulmonary malformations is this unique combination: proximal obliteration with preserved distal parenchymal development. The isolated distal lung segment, deprived of central airway communication, continues alveolar development but becomes dependent on collateral air drift from adjacent functional airways via the pores of Kohn (interalveolar communications) and canals of Lambert (bronchioloalveolar communications) [5,15]. This developmental anomaly creates characteristic pathological features: the obstructed bronchus becomes dilated and filled with mucus, forming a bronchocele, while the isolated distal parenchyma develops regional hyperinflation (emphysema) due to air trapping through collateral ventilation. The combination of mucus-filled bronchocele and adjacent emphysematous lung creates the pathognomonic radiological appearance, a finger-like or tubular opacity extending from the hilum with surrounding hyperlucent lung [6,7].

This case illustrates several important clinical and diagnostic considerations regarding congenital bronchial atresia presenting in adulthood with recurrent respiratory infections. The anatomical location in our patient, the left lower lobe, represents an unusual presentation, as the apicoposterior segment of the left upper lobe is affected in approximately 50% of cases, with lower lobe involvement occurring in only 15% of cases bilaterally [6,7]. This atypical distribution emphasizes that clinicians should maintain diagnostic vigilance for structural bronchial anomalies even when presentations deviate from classical patterns. While the distal lung segment beyond the atretic bronchus is theoretically isolated from central airways, recurrent respiratory infections occur in up to 21% of adult patients [11,12]. This apparent paradox challenges the assumption of sterility in the isolated segment. The same collateral ventilation pathways (pores of Kohn and channels of Lambert) that permit air entry and cause characteristic regional emphysema may also serve as routes for bacterial translocation or microaspiration from adjacent lung segments [15]. Furthermore, ineffective mucus clearance despite theoretical isolation, combined with regional emphysema and architectural distortion, may compromise local immune defenses and create a microenvironment susceptible to bacterial colonization [15]. In our patient, additional predisposing factors included underlying asthma with chronic inhaled corticosteroid use, which may cause local immunosuppression and impair innate immune responses in the respiratory mucosa [16,17]. Of particular interest was the temporal relationship between COVID-19 infection and the subsequent development of recurrent pneumonia, suggesting a potential role for antecedent viral infection as a triggering factor in this previously asymptomatic anatomical variant.

This hypothesis is supported by emerging evidence that SARS-CoV-2 causes persistent airway inflammation, epithelial damage, and altered mucociliary clearance lasting weeks to months post-infection [17,18]. In a patient with underlying bronchial atresia, this post-COVID inflammatory milieu may have precipitated symptomatic disease in a previously asymptomatic anatomical variant, with impaired immunity, damaged epithelial barriers, and pre-existing structural abnormality creating conditions favorable for recurrent bacterial superinfection. A critical teaching point is the importance of appropriately timed radiological reassessment following community-acquired pneumonia. Current guidelines recommend follow-up chest radiography 6-8 weeks after pneumonia in high-risk patients [19], yet even in younger patients without traditional risk factors, persistent or recurrent infections in the same anatomical location should prompt systematic investigation for structural abnormalities. Initial CT during active infection revealed only consolidation, obscuring the underlying anomaly. Only after complete clinical and radiological resolution did the characteristic features, mucus-filled bronchocele and regional emphysema, become clearly apparent. This underscores that timing is critical: imaging performed too early may miss subtle anatomical abnormalities masked by inflammation, while delayed or absent follow-up may leave underlying pathology undiagnosed.

Management of bronchial atresia remains debated. Conservative management with radiological surveillance is generally recommended for asymptomatic individuals, while surgical resection is typically reserved for recurrent severe infections, chronic refractory symptoms, or when malignancy cannot be excluded [14,20]. In our patient, several factors supported conservative management: complete resolution with medical therapy, absence of severe recurrent symptoms requiring repeated hospitalizations, well-controlled asthma, and the benign natural history in most adults. The multidisciplinary consensus was that watchful waiting with regular clinical and radiological follow-up represented the most appropriate strategy.

## Conclusions

This case of CBA presenting with recurrent pneumonia in an adult highlights several important clinical insights. Structural bronchial anomalies should be considered in recurrent localized respiratory infections, even in adults without prior symptoms, and appropriately timed post-infectious radiological reassessment is crucial for identifying underlying anatomical abnormalities that may be obscured during active infection. Bronchial atresia can present outside its typical anatomical distribution, emphasizing the need for diagnostic vigilance regardless of location.

This case illustrates the complex interplay between congenital anatomical variants, acquired risk factors (asthma, inhaled corticosteroids), and infectious triggers (COVID-19) in precipitating symptomatic disease. Given the rarity of this anomaly and its frequently asymptomatic nature, only isolated case reports and small case series exist in the literature, with no established guidelines for standardized management approaches. Consequently, therapeutic decisions must be individualized and discussed in a multidisciplinary setting, weighing patient-specific factors such as symptom severity, recurrence patterns, and underlying comorbidities. Publication and dissemination of such cases remain essential to raise awareness within the medical community about this uncommon congenital anomaly and to progressively expand our collective understanding of its natural history, clinical presentations, and optimal management strategies across diverse patient populations.
